# Liposomes Loaded With Phosphatidylinositol 5-Phosphate Improve the Antimicrobial Response to *Pseudomonas aeruginosa* in Impaired Macrophages From Cystic Fibrosis Patients and Limit Airway Inflammatory Response

**DOI:** 10.3389/fimmu.2020.532225

**Published:** 2020-10-02

**Authors:** Noemi Poerio, Federica De Santis, Alice Rossi, Serena Ranucci, Ida De Fino, Ana Henriquez, Marco M. D’Andrea, Fabiana Ciciriello, Vincenzina Lucidi, Roberto Nisini, Alessandra Bragonzi, Maurizio Fraziano

**Affiliations:** ^1^ Dipartimento di Biologia, Università degli Studi di Roma “Tor Vergata”, Roma, Italy; ^2^ Unità di Infezioni e Fibrosi Cistica, Istituto Scientifico San Raffaele, Milano, Italy; ^3^ Unità Operativa Complessa Fibrosi Cistica, Dipartimento di Medicina Pediatrica, Ospedale Pediatrico Bambino Gesù, Roma, Italy; ^4^ Dipartimento di Malattie Infettive, Istituto Superiore di Sanità, Roma, Italy

**Keywords:** phosphatidylinositol 5-phospate, host-directed therapy, cystic fibrosis, innate immunity, *Pseudomonas aeruginosa*, liposome

## Abstract

Despite intensive antimicrobial and anti-inflammatory therapies, cystic fibrosis (CF) patients are subjected to chronic infections due to opportunistic pathogens, including multidrug resistant (MDR) *Pseudomonas aeruginosa.* Macrophages from CF patients show many evidences of reduced phagocytosis in terms of internalization capability, phagosome maturation, and intracellular bacterial killing. In this study, we investigated if apoptotic body-like liposomes (ABLs) loaded with phosphatidylinositol 5-phosphate (PI5P), known to regulate actin dynamics and vesicular trafficking, could restore phagocytic machinery while limiting inflammatory response in *in vitro* and *in vivo* models of MDR *P. aeruginosa* infection. Our results show that the *in vitro* treatment with ABL carrying PI5P (ABL/PI5P) enhances bacterial uptake, ROS production, phagosome acidification, and intracellular bacterial killing in human monocyte-derived macrophages (MDMs) with pharmacologically inhibited cystic fibrosis transmembrane conductance regulator channel (CFTR), and improve uptake and intracellular killing of MDR *P. aeruginosa* in CF macrophages with impaired bactericidal activity. Moreover, ABL/PI5P stimulation of CFTR-inhibited MDM infected with MDR *P. aeruginosa* significantly reduces NF-κB activation and the production of TNF-α, IL-1β, and IL-6, while increasing IL-10 and TGF-β levels. The therapeutic efficacy of ABL/PI5P given by pulmonary administration was evaluated in a murine model of chronic infection with MDR *P. aeruginosa*. The treatment with ABL/PI5P significantly reduces pulmonary neutrophil infiltrate and the levels of KC and MCP-2 cytokines in the lungs, without affecting pulmonary bacterial load. Altogether, these results show that the ABL/PI5P treatment may represent a promising host-directed therapeutic approach to improve the impaired phagocytosis and to limit the potentially tissue-damaging inflammatory response in CF.

## Introduction

Cystic fibrosis (CF) is an autosomal recessive genetic disease caused by a mutation in the gene encoding the cystic fibrosis transmembrane conductance regulator channel (CFTR) (1). The CFTR is usually expressed on the apical membrane of epithelia, and its dysfunction causes a defective chloride secretion leading to a modification in the airway surface liquid (2). The pathophysiological changes in CF result in a systemic disease, which affects the pancreas, liver, reproductive tract, and mainly the lungs (3). Here, the loss of function of CFTR causes a defective mucociliary clearance and a dramatic production of sticky mucus, which is associated with chronic infection by opportunistic pathogens, such as *P. aeruginosa* (4). Infections sustained by MDR *P. aeruginosa* in CF are increasing, reflecting cumulative exposure to antibiotic treatment (5). Moreover, the chronic bacterial infections associated with the persistent inflammation, leading to pulmonary insufficiency, represent the main cause of mortality and morbidity in CF patients (6). Today, the identification of novel host- and/or pathogen-directed therapeutic tools represents an urgent challenge for the scientific community to fight the emergence of MDR pathogens, as well as a priority also at the global level.

The defective antimicrobial response exerted by innate immune cells in CF patients has been documented and depends, at least in part, on a dysfunctional phagocytosis process (7, 8). Phagocytosis is an important innate effector mechanism deputed to the intracellular elimination of invading pathogen by the generation of highly microbicidal organelles called phagolysosomes. These organelles originate from a phagosome, generated by the invagination of plasma membrane, which matures to a fully microbicidal phagolysosome, through sequential events of fusion with early endosomes, late endosomes, and, ultimately, lysosomes. This process is driven by a topologically and timely coordinated expression of second lipid messengers, which recruit signal proteins, on the nascent or maturing phagosome, through specific lipid-binding domains (9, 10), and may be target of bacterial interference (11).

The second lipid messenger phosphatidylinositol 5-phosphate (PI5P) is a minor phosphoinositide representing less than 10% of the total lipids (12). PI5P can be directly produced from phosphatidyl inositol **(**PI**)** by the activity of phosphoinositide 5-kinase (PIKfyve) or by the dephosphorylation of phosphatidylinositol 3,5-bisphosphate (PI3,5P_2_) by mytubularin 3-phosphatases (13). PI5P is present at the cellular membrane and at the early phagosome (14), and its level result increased during the late stages of the phagocytosis process (15). Moreover, it can regulate endosome vesicle trafficking (16), cellular actin remodeling, and bacterial invasion (14), and can be involved in class III phosphatidylinositol 3-kinase (Vps34)-independent autophagy activation (17).

In this study, we have generated asymmetric apoptotic body-like liposomes (ABLs) composed by phosphatidylserine (PS) at the outer membrane surface resembling an apoptotic body, to target macrophages and to downmodulate inflammatory reaction (18), and by the bioactive lipid PI5P at the inner membrane surface to enhance the phagocytosis process. In particular, this study evaluates the immunotherapeutic value of ABL/PI5P *in vitro* in impaired macrophages from CF patients and *in vivo* in models of *P. aeruginosa* infection, assessed in terms of i) uptake and intracellular bacterial killing, ii) mechanisms of bactericidal activity, and iii) potentially tissue-damaging inflammatory response.

## Material and Methods

### Liposome Preparation

Apoptotic body-like liposomes (ABLs) were produced as previously described (19). Briefly, the inner monolayer lipids composed by 1,2-dioleoyl-*sn*-glycero-3-phospho-(1′-myo-inositol-5′-phosphate) (PI5P, Avanti Polar Lipids) were suspended in anhydrous dodecane (Sigma) at a concentration of 0.05 mg/ml. L-α-phosphatidylserine (PS, Avanti Polar Lipids) was used as outer monolayer lipid and was added to a 99:1 dodecane:silicone solution to obtain a final concentration of 0.05 mg/ml. Asymmetric liposomes were prepared by adding 2 ml of outer monolayer lipid suspension over 3 ml of cell culture medium (for *in vitro* experiments) or saline (for *in vivo* experiments). Finally, 100 μl of the inner monolayer lipid suspensions were added over the 2-ml lipid phase, and the samples were centrifuged at 120 × *g* for 10 min. After the centrifugation, ABLs were collected in the aqueous phase using a 5-ml syringe with a 16-gauge stainless steel needle, in order to produce PS outside/PI5P inside liposomes (ABL/PI5P). Liposomes were then quantified by a flow cytometer FACSCalibur (Becton Dickinson), allowing quantification of monodispersed vesicles >0.2 μm in diameter.

### Cell Culture

Primary monocyte-derived macrophages (MDMs) were prepared as previously described (17). Briefly, peripheral blood mononuclear cells (PBMCs) from healthy donors and CF patients were isolated by Ficoll density gradient, and monocytes were then positively sorted using anti-CD14 monoclonal antibodies conjugated to magnetic microbeads (Miltenyi Biotec), according to manufacturer’s instructions. Monocytes were then suspended in complete medium and incubated for a further 5 days in 96-well plates at a concentration of 10^6^ cells/ml in the presence of M-CSF (50 ng/ml, Miltenyi Biotec) to get differentiated macrophages.

### Bacteria

MDR *P. aeruginosa* strain (ATCC^®^ BAA-2113) was used in *in vitro* experiments and MDR-RP73 *P. aeruginosa* clinical isolate (20) was used in an *in vivo* mouse model of chronic *P. aeruginosa* infection (21, 22). The BAA-2113 single colony was collected by streaking on Trypticase soy agar (TSA, BD Difco™) and then suspended in 15 ml of Trypticase soy broth (TSB, BD Difco™). Bacteria were grown in Erlenmeyer flask at 37°C under stirring for 18 h, and their growth was monitored by measuring the optical density at a wavelength of 600 nm by Varioskan LUX Multimode Microplate Reader (Thermo Fisher Scientific). BAA-2113 was stored at −80°C until use after suspension in TSB and 30% glycerol.

For *in vivo* experiments, an aliquot of RP73 strain from glycerol stocks (TSB + 25% glycerol) was streaked for isolation on TSA and incubated at 37°C O/N. One colony was picked from the plate and used to inoculate 10 ml of TSB and placed overnight in a shaking incubator at 37°C 200 rpm. Thereafter, bacterial suspension was diluted to 0.15 OD/ml in 20 ml of TSB/flask and grown for 4 h at 37°C at 200 rpm, to reach the log phase.

### Patients

CF patients (n = 19) were enrolled at “Bambino Gesù” Children’s Hospital in Rome, Italy. All of the CF patients were clinically stable at the time of blood donation (5 ml). Controls (n = 20) were represented by buffy coats from healthy blood donors, attending at the Blood Transfusion Unit of Policlinico “Umberto I” in Rome, Italy. Clinical and demographic features of CF patients as well as healthy controls are summarized in **Table 1**.

**Table 1:** Demographic and clinical characteristics of cystic fibrosis (CF) patients and healthy donors (HD).

CF	Age (range)	Genotype	Microbiology	FEV-1 (%)	HD	Age (range)
1	26–30	F508del/N1303K	*S.a*, *A.x.*, *S.m.*	44	1	36–40
2	16–20	F508del/P5L	*S.a.*	79	2	21–25
3	11–15	G576A/R668C	*S.a*, *En.c.*, *E.a.*	106	3	51–55
4	21–25	F508del/F508del	*S.a.*, *B.b.*	85	4	56–60
5	31–35	F508del/f508del	*S.a.*, *Bu.c.*	72	5	41–45
6	21–25	F508del/621+1G>T	*P.a.*, *Bu.c.*	50	6	36–40
7	41–45	F508del/F508del	*S.m.*, *Bu.c.*	91	7	56–60
8	31–35	F508del/W1282X	*S.a.*, *P.a.*	109	8	56–60
9	26–30	F508del/F508del	*S.a.*, *Es.c.*	115	9	41–45
10	21–25	F508del/G1244E	*S.a.*	97-103	10	56–60
11	26–30	F508del/G542X	*E.f.*, *S.a.*, *C.g.*, *S.m.*	65	11	51–55
12	26–30	DeltaF508/G85E	*S.a.*, *P.m.*, *S.m.*, *A.f.*	82	12	46–50
13	6–10	DeltaF508/R334L	*S.p.*, *S.a.*, *H.i.*	95-104	13	21–25
14	11–15	R553X/3272-26A->G	*S.a.*	88	14	41–45
15	6–10	G85E/621+1G->T	*A.f.*, *H.i.*	86	15	56–60
16	21–25	G1244E/T338I	*H.i.*	102	16	51–55
17	6–10	1717-1G->A/E831X	GAS, *Br.c.*, *H.i.*	119	17	41–45
18	16–20	W1282X/2789+5G>A	*S.a.*, *Sc.a.*	85	18	31–35
19	26–30	DeltaF508/DeltaF508	*S.a.*, *P.a.*	25	19	21–25
					20	26–30

S.a., Staphylococcus aureus; A.x., Achromobacter xylosoxidans; S.m., Stenotrophomonas maltophilia; En.c., Enterobacter cloacae; E.a., Enterobacter asburiae; B.b., Bordetella bronchiseptica; Bu.c., Burkholderia cepacia; P.a., Pseudomonas aeruginosa; Es.c., Escherichia coli; E.f., Enterococcus faecalis; C.g., Candida glabrata; P.m., Proteus mirabilis; A.f., Aspergillus fumigatus; S.p., Streptococcus pneumoniae; H.i., Haemophlus influenzae; GAS, Group A Streptococcus; Br.c., Branhamella catarrhalis; S.a., Scedosporium apiospermum.

### Evaluation of *In Vitro* Bacterial Uptake and Intracellular Growth

To assess bacterial uptake, MDMs from healthy donors or from CF patients were distributed in 96-well plates at a concentration of 2 × 10^5^ cells/well and were stimulated with ABL/PI5P used at a ratio of 1:1 (ABL:MDM), for 30 min before infection and/or simultaneously with the infection, in the presence or absence of the CFTR inhibitor INH172 (Sigma), used at a concentration of 10 µM. Then cells were washed once and infected with MDR *P. aeruginosa* for 1 h at 37°C at an MOI of 30 in the presence or absence of INH172. Thereafter, extracellular bacilli were killed at 1 h of incubation with 400 µg/ml amikacin. Finally, cells were lysed with 1% deoxycholate (Sigma), samples were diluted in PBS-tween 80, and colony-forming units (CFUs) were quantified by plating bacilli in triplicate on TSA.

To assess intracellular bacterial growth, MDMs from healthy donors or from CF patients were distributed in 96-well plates at a concentration of 2 × 10^5^ cells/well and were infected with MDR *P. aeruginosa*, for 1 h at 37°C at an MOI of 30, in the presence or absence of INH172, used at a concentration of 10 µM. Thereafter, extracellular bacilli were killed at 1 h of incubation with 400 µg/ml amikacin. Cells were then washed and incubated with ABL/PI5P, added to a ratio of 1:1 (ABL:MDM) for a further 2 h, in the presence or absence of INH172. Finally, cells were lysed with 1% deoxycholate (Sigma), samples were diluted in PBS-tween 80, and CFUs were quantified by plating bacilli in triplicate on TSA. In order to evaluate the role of ROS and of phagosome acidification in intracellular bacterial killing, *P. aeruginosa*-infected cells were treated simultaneously with ABL/PI5P with either PEG-Catalase (100 U/ml) or Concanamycin A (10 nM), respectively.

### Fluorimetric Analysis

Phagosome acidification was assessed by using the fluorescent probe Lysosensor green DND 189 (Molecular Probes) (23), which measures the pH of acidic organelles, such as phagolysosomes. Briefly, MDM from healthy donors were pretreated or not for 1 h with 10 µM INH172 and then exposed or not to Crimson fluorescent microbeads (1 µm FluoSpheres^®^carboxylate-modified microspheres, LifeThechnologies), for 1 h at 37°C at a ratio of 5:1 in the presence or absence of 10 µM of INH172, in order to exclude possible differences in microbead internalization among experimental groups. Then cells were washed and incubated for a further 90 min with ABL/PI5P, added to a ratio of 1:1 (ABL:MDM), in the presence or absence of INH172. Cells were stained for 15 min at 37°C with 1 µM of Lysosensor green DND 189. pH calibration curve was obtained by incubating macrophages in calibration buffers at pH 4.5, 5.5, 6.5, and 7.5 (Intracellular pH Calibration Buffer Kit, Molecular Probes), and by labeling cells for 15 min at 37°C with 1 µM of Lysosensor green DND 189 according to the manufacturer’s instructions. pH was evaluated by fluorometry by setting the wavelength of excitation at 443 or 625 nm and emission at 505 or 645 nm, for Lysosensor green DND 189 and Crimson fluorescent microbeads, respectively.

ROS generation was analyzed by loading MDM isolated from healthy donors with the fluorescent indicator 20,70-dichlorofluorescein diacetate (DCF, Molecular Probes), used at a concentration of 10 μM, for 40 min at 37°C in the dark. Thereafter, MDM isolated from healthy donors were pretreated or not for 1 h with 10 µM INH172 and then exposed or not to Crimson fluorescent microbeads (1 µm FluoSpheres^®^ carboxylate-modified microspheres, Life Technologies), for 1 h at 37°C at a ratio of 5:1 in the presence or absence of 10 µM of INH172, in order to exclude possible differences in microbead internalization among experimental groups. Cells were then washed and incubated for a further 90 min in the presence or absence of INH172 with ABL/PI5P, added to a ratio of 1:1 (ABL:MDM). The production of ROS was evaluated by fluorometry by setting the wavelength of excitation at 443 or 625 nm and emission at 505 or 645 nm, for DCF and Crimson fluorescent microbeads, respectively. Fluorescence has been evaluated by the use of a Varioskan LUX Multimode Microplate Reader (Thermo Fisher Scientific).

### Mouse Model of Chronic Infection

Immunocompetent C57Bl/6NCrlBR male mice (8–10 weeks, Charles River) (n = 16 treated with 3 × 10^5^ ABL/PI5P and n = 16 treated with vehicle) were challenged with 3–4 × 10^5^ CFUs of the *P. aeruginosa* MDR-RP73 embedded in agar beads for chronic infection by intratracheal (i.t.) administration. Agar beads were prepared following established procedures (21, 24). Local treatment by Penn-Century MicroSprayer^®^ Aerosoliser with 3 × 10^5^ ABL/PI5P started soon (5 min) after infection and was repeated daily for 6 days. Body weight and health status were monitored daily. After 6 days postinfection, lung CFUs and cell counts in the bronchoalveolar lavage fluid (BALF) were analyzed as previously described (21, 24). Finally, 6 days after infection, murine lungs were excised aseptically and homogenized in 2 ml of PBS added with protease inhibitors (Complete™ Protease Inhibitor cocktail—Roche) using the homogenizer GentleMACS™ Octo Dissociator, and the levels of TNF-α, KC, JE, and MIP-2 in the supernatant of murine lungs were measured by ELISA kit (DuoSet^®^ ELISA Development Systems).

### Enzyme-Linked Immunosorbent Assay

MDMs were infected or not with *P. aeruginosa* (MOI 30) in the presence or absence of INH172 and stimulated or not with ABL/PI5P at a ratio of 1:1 (ABL:MDM) for 2 h. Thereafter, supernatants were collected, cells were lysed, and both stored at −20°C until analysis. The levels of tumor necrosis factor-α (TNF-α), interleukin-1β (IL-1β), IL-6, IL-10, and transforming growth factor-beta (TGF-β) in the supernatants of MDMs were measured by human TNF-α ELISA kit (BD Biosciences), human IL-6 DuoSet^®^ ELISA Development Systems, human IL-1β DuoSet^®^ ELISA Development Systems, human IL-10 DuoSet^®^ ELISA Development Systems, and human TGF-β DuoSet^®^ ELISA Development Systems (all by R&D system) and used according to the manufacturer’s instructions. The levels of murine TNF-α, KC, JE, and MIP-2 were measured by DuoSet^®^ ELISA Development Systems (R&D system). The activation of NF-κB transcription factor was assessed on lysed cells by “NFkB p65 (Total/Phospho) Human InstantOne™ ELISA Kit” (Invitrogen) and used according to the manufacturer’s instructions.

### Statistics

Comparison between groups was done using Student’s *t* test, as appropriate, for normally distributed data. The Wilcoxon rank test sum or Mann–Whitney test was performed for data that were not normally distributed.

### Ethics Statement

Buffy coats from anonymized healthy donors, who gave their written informed consent to donate the nonclinically usable components of their blood for scientific research, were obtained from the Blood Transfusion Unit of Policlinico “Umberto I” in Rome. The present study, which is based on nonclinical *in vitro* research, did not require any specific approval from an ethical committee, according to the Italian law (decree by Ministero della Salute by February 8, 2013, published on Gazzetta Ufficiale della Repubblica Italiana no. 96 of April 24, 2013, and legislative decree no. 211 of June 24, 2003, published on Gazzetta Ufficiale della Repubblica Italiana no. 184 of August 9, 2003). Cystic fibrosis patients, giving their (or parental) informed consent to participate in the study, were enrolled at “Bambino Gesù” Children’s Hospital in Rome after having received detailed information on the scope and objectives of the study by a sanitary personnel who explained the patient information leaflet (ethics approval #738/2017 of “Bambino Gesù” Children’s Hospital, Rome).

Animal studies adhered to the Italian Ministry of Health guidelines for the use and care of experimental animals (IACUC #733).

Research with *P. aeruginosa* RP73 clinical isolate from CF patient has been approved by the Ethics Commission of Hannover Medical School, Germany. The patient and parent gave informed consent before the sample collection.

## Results

### ABL Loaded With PI5P Improve Dysfunctional Bacterial Uptake in CF and INH172 Treated Macrophages

**Figure 1:**
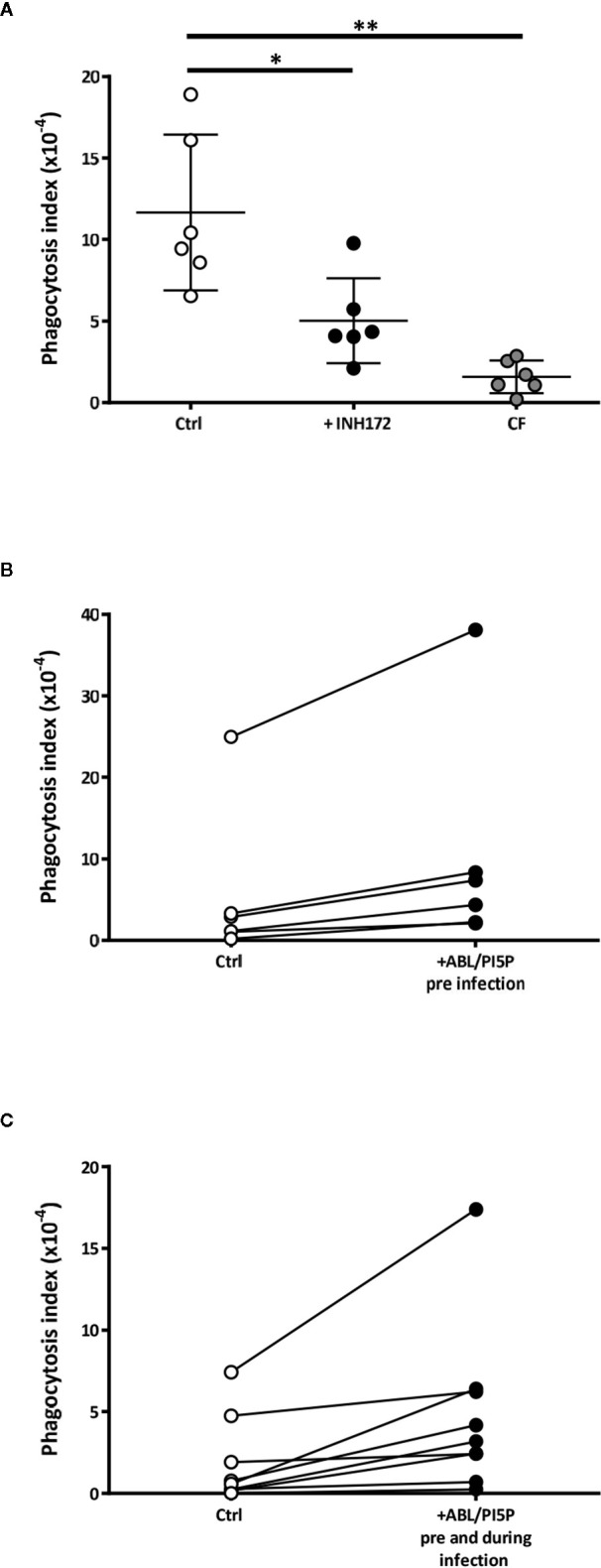
Dysfunctional *Pseudomonas aeruginosa* uptake in macrophages with pharmacologically inhibited or naturally mutated cystic fibrosis transmembrane conductance regulator channel (CFTR) and its enhancement by apoptotic body-like liposome/phosphatidylinositol 5-phosphate (ABL/PI5P) stimulation. **(A)** Monocyte-derived macrophages (MDMs) from healthy donors, treated or not with INH172, or from cystic fibrosis (CF) patients were infected with multidrug-resistant (MDR) *P. aeruginosa* (BAA-2113 strain) at an MOI of 30. **(B, C)** CF MDMs were stimulated or not with ABL/PI5P for 30 min before infection **(B)** or before and during infection **(C)**. Cells were then infected with MDR *P. aeruginosa* (BAA-2113 strain) at an MOI of 30. The bacterial uptake was quantified by colony-forming unit (CFU) assay and indicated as phagocytosis index, calculated as the ratio between the CFUs obtained immediately after the infection and the inoculum. **(A)** Statistical analysis was performed by using the two-sided Mann–Whitney test and *p < 0.05; **p < 0.01 in comparison with control cells (healthy donors, n = 6; CF patients, n = 6). **(B, C)** Statistical analysis was performed by using the two-sided Wilcoxon rank sum test (**B**, n = 6) p = 0.03 and (**C**, n = 9) p = 0.004.

CF macrophages show defective *P. aeruginosa* internalization (25–27). Hence, we tested the capability of ABL carrying PI5P to improve phagocytosis of MDR *P. aeruginosa* in macrophages with disabled CFTR. Results confirmed that the bacterial uptake of MDM from CF patients or INH172-treated MDM from healthy donors was dysfunctional compared to that of untreated MDM (**Figure 1A**). The dysfunctional bacterial uptake capacity was significantly improved by the preventive treatment with ABL/PI5P of INH172-treated dTHP-1 cells, infected with MDR *P. aeruginosa* at an MOI of 30 and 10, and resulted completely restored at an MOI of 30 (**Figure S1A**). Moreover, this effect was specific for ABL/PI5P, as any effect was not observed when liposomes composed by either PS or PI5P only were used (**Figure S1B**). Bacterial internalization was also improved by the pretreatment with ABL/PI5P of primary MDM, with pharmacologically inhibited CFTR (**Figure S2**), and of CF MDM (**Figures 1B, C** **)**. No modification of the bacterial uptake was observed when ABL/PI5P was used simultaneously with MDR *P. aeruginosa* infection, excluding that liposomes exerted their effect interacting with the pathogen (**Figure S2**).

### Treatment With ABL/PI5P Rescues Impaired Phagosome Maturation and ROS Generation in Macrophages With Pharmacologically Inhibited CFTR

**Figure 2:**
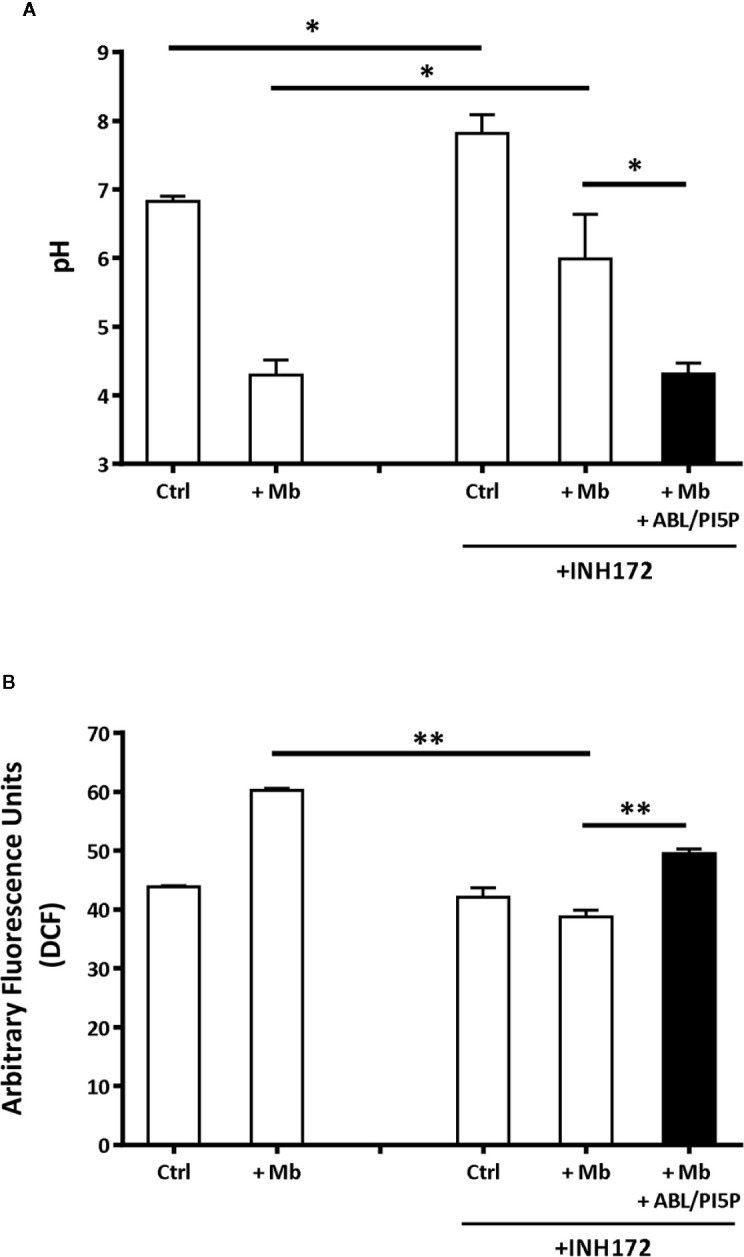
Treatment with ABL/PI5P rescues impaired phagosome acidification and ROS production in MDM with pharmacologically inhibited CFTR. Primary MDMs, treated or not with INH172, were exposed for 1 h to 1-µm microbeads (Mb). Phagosomal pH **(A)** and ROS **(B)** were assessed, by staining with Lysosensor green DND 189 or DCF, after 90 min of stimulation with ABL/PI5P, respectively. Results are shown as mean + standard deviation of the values obtained from triplicate cultures and are representative of experiments with cells by two different donors. *p < 0.05; **p < 0.01, in comparison with INH172-unstimulated cells and in comparison with untreated cells by one-sided Student’s t test.

Dysfunctional activity of CFTR leads to impaired phagosome maturation due to unbalanced influx of chloride ions (Cl^−^) that does not allow intraphagosomal acidification (8). In this context, we determined basal intracellular pH and ROS production, both in the normal and CFTR-pharmacologically inhibited macrophages. MDMs with CFTR functionally inhibited by INH172 had a more basic intracellular pH than untreated MDM and, after exposure to microbeads, showed an impaired phagosome acidification (**Figure 2A**), which could be completely restored after 90 min of treatment with ABL/PI5P (**Figure 2A**). This result was confirmed by using microbeads labeled with NHS, a pH-sensitive fluorochrome, whose fluorescence decreases proportionally to acidification of phagosome microenvironment: MDMs with CFTR functionally inhibited by INH172 and treated with ABL/PI5P showed a reduction of NHS fluorescence at levels comparable to that of control MDMs (**Figure S3**).

Phagosome acidification and ROS generation are sequential steps leading to intracellular bacterial killing and type II NADPH oxidase (NOX-2) assemblies from component subunits on maturing phagosomes (28). On these grounds, we monitored ROS generation in MDM with or without pharmacologically inhibited CFTR following exposure to microbeads and after 90 min of treatment with ABL/PI5P. As expected, the exposure to microbeads induced a significant ROS generation in control cells (**Figure 2B**). On the contrary, the exposure to microbeads provoked an impaired ROS production in MDM with INH172-inhibited CFTR, which was significantly restored by the ABL/PI5P treatment (**Figure 2B**). Together, these results show that the inhibition of CFTR by INH172 causes an impaired phagosome acidification and a reduced ROS production that could be significantly recovered by the treatment with ABL/PI5P.

### ABL/PI5P Promote Intracellular Bacterial Killing of INH172-inhibited Control Macrophages and CF Macrophages

**Figure 3:**
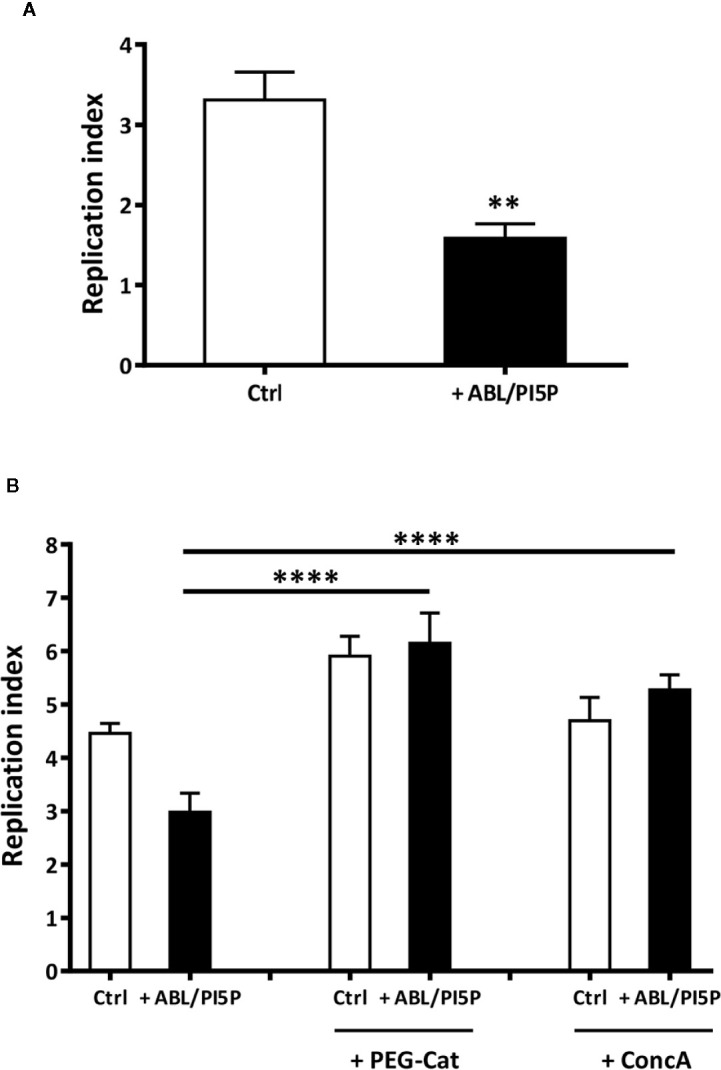
ABL/PI5P promotes both ROS and phagolysosome acidification-dependent intracellular *P. aeruginosa* killing in MDM with pharmacologically inhibited CFTR. **(A)** Primary MDMs were exposed to the CFTR inhibitor INH172 at a concentration of 10 µM, infected with MDR *P. aeruginosa* (BAA-2113 strain) and then stimulated for further 2 h with ABL/PI5P. **(B)** Primary MDMs were exposed to the CFTR inhibitor INH172 at a concentration of 10 µM, infected with MDR *P. aeruginosa* (BAA-2113 strain), and then stimulated for a further 2 h with ABL/PI5P in the presence or absence of catalase (PEG-Cat) or Concanamycin A (Conc A), at a concentration of 100 U/ml or 10 nM, respectively. Bacterial growth was assessed by CFU assay, and replication index was calculated as the ratio between the CFU obtained after 2 h of infection, in the presence or absence of ABL/PI5P, and the CFU was obtained before the addition of liposomes. The results are shown as mean + standard deviation of the values obtained from triplicate of each condition. **p < 0.01; ****p < 0.0001 by two-sided Student’s test.

Since ABLs/PI5Ps were shown to restore the functional intraphagosomal acidification and oxidative burst of macrophages with pharmacologically inhibited CFTR, we investigated whether an increased bactericidal activity against MDR *P. aeruginosa* strains could also represent a functional consequence of ABL/PI5P treatment of cells with altered CFTR function. In this context, we preliminarily tested the capability of ABL/PI5P to improve intracellular bacterial killing in dTHP-1 cells with disabled CFTR infected with MDR *P. aeruginosa* (BAA-2113 strain) at the MOI of 30 and 10. Results expressed in **Figure S4A** show a significant reduction in intracellular bacterial viability after exposure to ABL/PI5P, which was higher at an MOI of 30. Moreover, such effect was specific for ABL/PI5P, as any effect was not observed when liposomes composed by either PS or PI5P only were used (**Figure S4B**). Thereafter, we investigated the effect of ABL/PIP5 on primary MDMs with pharmacologically inhibited CFTR. Our results show that 2 h of ABL/PI5P treatment on INH172-treated MDM significantly enhances the intracellular killing of MDR *P. aeruginosa* strain (BAA-2113) (**Figure 3A**) as well as of a panel of additional three MDR *P*. *aeruginosa* strains (BAA-2108, BAA-2111, and BAA-2112) (**Figure S5**).

In order to evaluate the role of phagosome acidification and/or of ROS generation in intracellular killing of MDR *P. aeruginosa* induced by ABL/PI5P, we exposed *P. aeruginosa-*infected cells to either Concanamycin A (ConcA), a specific inhibitor of V-ATPases blocking phagosome acidification, or polyethylene glycol-Catalase (PEG-Cat), which reduces hydrogen peroxide to water. Results show that intracellular killing of MDR *P. aeruginosa*, induced by ABL/PI5P stimulation of MDM with pharmacologically inhibited CFTR, is ROS mediated and phagosome acidification dependent, as it results ineffective in the presence of Peg-Cat and Conc A, respectively (**Figure 3B**).

**Figure 4:**
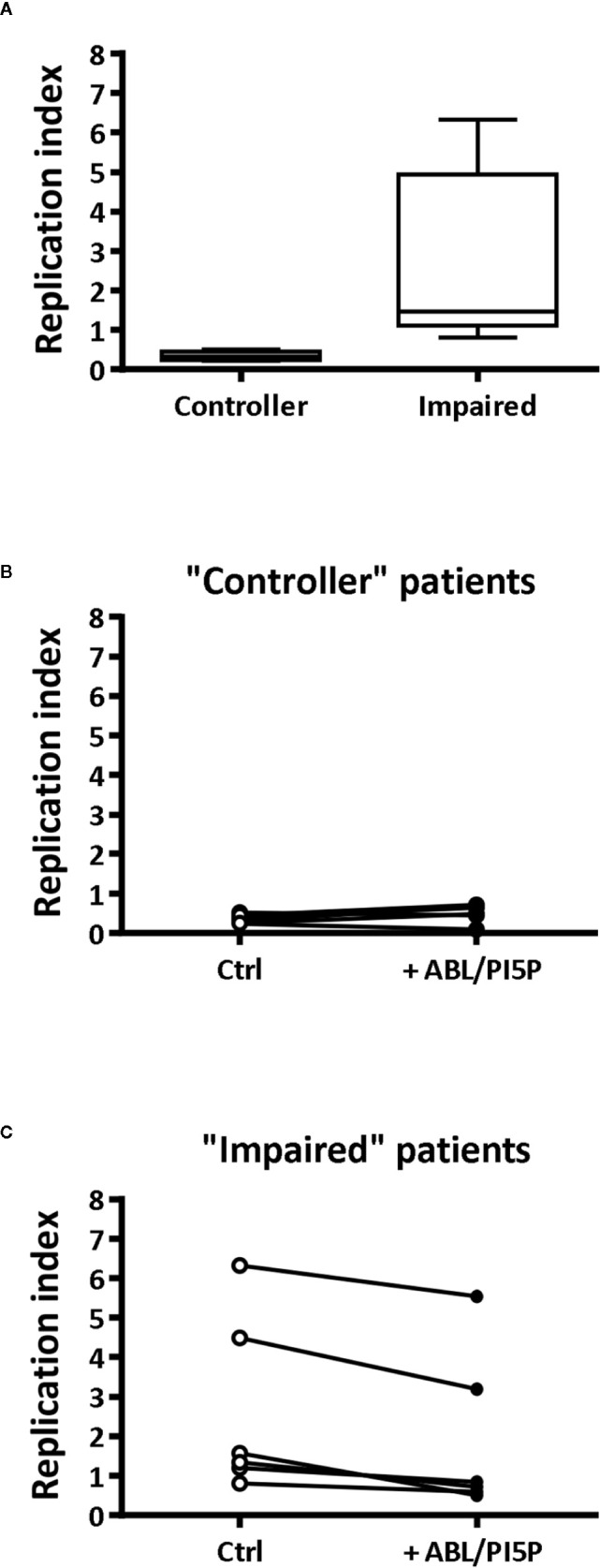
ABL/PI5P enhances intracellular bacterial killing in CF macrophages characterized by impaired antimicrobial activity. MDM isolated from CF patients (n = 12) were infected with MDR *P. aeruginosa* (BAA-2113 strain) and then stimulated for another 2 h with ABL/PI5P. Bacterial growth was assessed by CFU assay, and replication index was calculated as the ratio between the CFU obtained after 2 h of infection in the presence or absence of ABL/PI5P and the CFU obtained before the addition of liposomes. **(A)** CF patients have been defined as “functional” or “controller” on the basis of bacterial replication index, less or higher than 1, respectively. Bacterial replication index is shown in “controller” (**B**, n = 6) and “impaired” (**C**, n = 6) macrophages from CF patients following ABL/PI5P stimulation. Statistical analysis was performed by using the two-sided Mann–Whitney test **(A)** and two-sided Wilcoxon matched-pairs signed rank test **(B, C)**. **(A)** p = 0.0022; **(B)** p = not significant; **(C)** p = 0.0313.

Finally, we tested the efficacy of ABL/PI5P in MDMs from CF patients. On the basis of the efficacy of freshly isolated and nontreated CF macrophages to limit intracellular bacterial growth, we could divide patients in two groups: “impaired” and “controller,” according to intracellular bacterial replication index higher or lower than 1, respectively (**Figure 4A**). Notably, MDM isolated from patients of the “impaired” group were susceptible to ABL/PI5P stimulation (**Figure 4C**), increasing significantly their intracellular killing upon liposome treatment, whereas ABL/PI5P did not further increase the intracellular killing of MDM isolated from patients belonging to the “controller” group (**Figure 4B**) or from healthy donors (**Figure S6**).

### ABL/PI5P Treatment Modulates Anti- and Pro- Inflammatory Cytokine Production in Macrophages With Pharmacologically Inhibited CFTR

**Figure 5:**
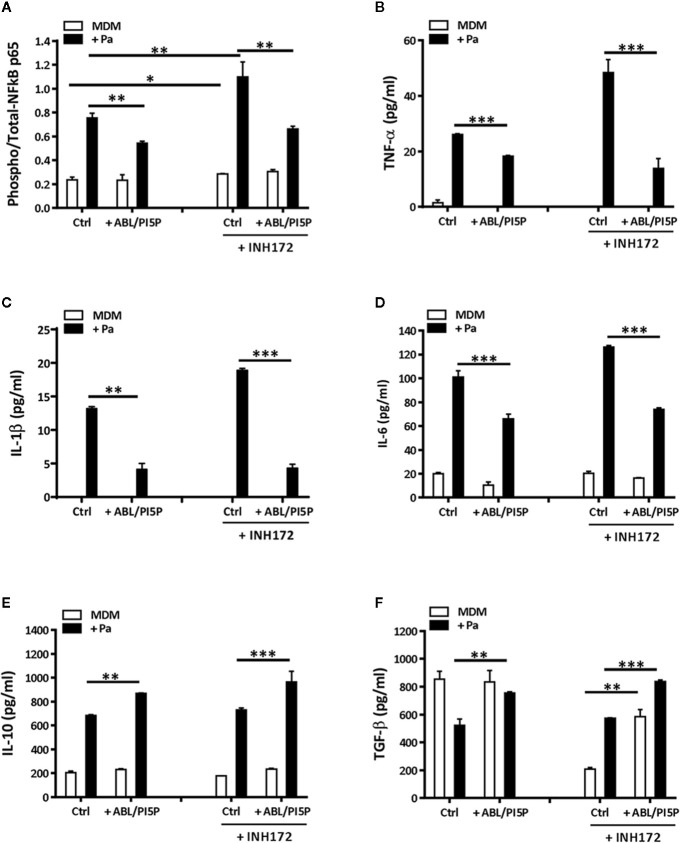
ABL/PI5P stimulation modulates NF-κB and cytokine production in MDM with pharmacologically inhibited CFTR. MDMs were treated or not with INH172, infected or not with MDR *P. aeruginosa* (Pa, BAA-2113 strain), and then stimulated or not with ABL/PI5P for 2 h. Thereafter, cells were lysed **(A)** or supernatants were collected **(B–F)**, and both were stored at −20°C until analysis. **(A)** Cell lysates were analyzed by NF-κB p65 (Total/Phospho) Human InstantOne™ ELISA kit, and results are shown as the ratio between phosphorylated and total NF-κB p65. The production of TNF-α **(B)**, IL-1β **(C)**, IL-6 **(D)**, IL-10 **(E)**, and TGF-β **(F)** was analyzed by ELISA. The results are shown as mean + standard deviation of the values obtained from triplicate of each conditions and are representative of experiments with cells from at least three different donors. *p < 0.05; **p < 0.01; ***p < 0.001 one-sided *t* test.

Chronic infection, mainly due to *P. aeruginosa*, and unresolved acute inflammation are key mechanisms responsible for progressive lung destruction in CF (29) and an effective host-directed therapeutic strategy should also limit the inflammation-based immunopathology. On the basis of previous results showing the anti-inflammatory effect of ABL (18), we wanted to investigate the effect of ABL/PI5P treatment of MDM incubated or not with INH172 on NF-κB activation and on the production of a panel of pro- and anti-inflammatory cytokines after infection with MDR *P. aeruginosa*. In this model, we could show high basal levels of NF-κB activation after CFTR inhibition, which further increased following infection with MDR *P. aeruginosa*. Interestingly, the same NF-κB activation levels were significantly reduced by the treatment with ABL/PI5P (**Figure 5A**). The reduced activation of NF-κB was confirmed by the comparative *in vitro* measure of cytokines whose transcription depends upon NF-κB activity (TNF-α, IL-1β, and IL-6). In fact, infected macrophages with dysfunctional CFTR showed a significant increase in TNFα, IL-1β, and IL-6 secretion in comparison with control infected macrophages, and ABL/PI5P treatment reduced the levels of the same inflammatory cytokines in infected macrophages irrespective of CFTR inhibition (**Figures 5B–D**). On the contrary, the secretion of anti-inflammatory cytokines, such as IL-10 and TGF-β, was significantly increased in ABL-/PI5P-treated MDMs (**Figures 5E, F** **).**

### ABL/PI5P Therapeutic Treatment Reduces Inflammatory Reaction in a Murine Model of MDR *P. aeruginosa* Chronic Infection

**Figure 6:**
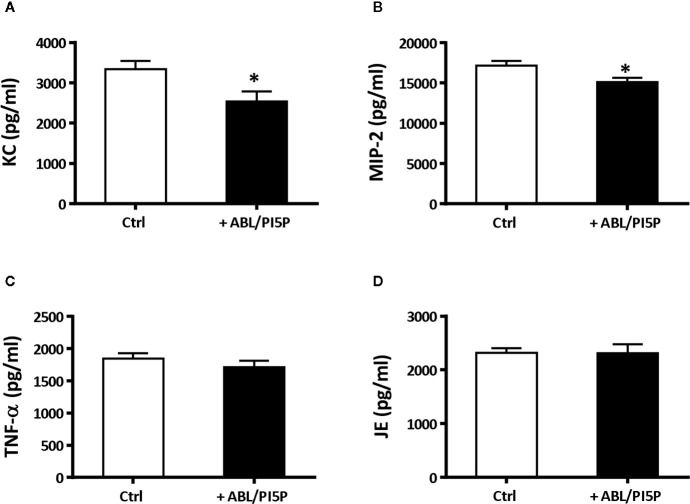
ABL/PI5P treatment modulates KC and MIP-2 production in a murine model of MDR *P. aeruginosa* chronic lung infection. C57Bl/6NCrlBR mice were infected with MDR *P. aeruginosa* (RP73 strain) and then treated with ABL/PI5P (n = 16) or vehicle (n = 16), as described in the *Material and Methods* section. At day 6 post-infection, mice were sacrificed. Levels of KC **(A)**, MIP-2 **(B)**, TNF-α **(C)**, and JE **(D)** upon treatment with ABL/PI5P or vehicle in the supernatants of lung homogenates were measured by ELISA. Data are shown as mean values + standard error. The data are pooled from two independent experiments. Statistical analysis was performed by using the two-sided Mann–Whitney test. Statistical significance is indicated: *p < 0.05. Outlier data, identified by Grubbs’ test, were excluded by the analysis.

**Figure 7:**
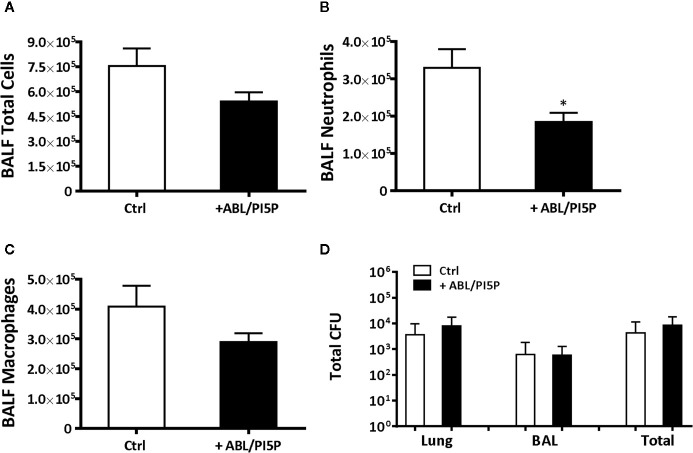
ABL/PI5P treatment reduces neutrophilic recruitment in a murine model of MDR *P. aeruginosa* chronic lung infection. C57Bl/6NCrlBR mice were infected with MDR *P. aeruginosa* (RP73 strain) and then treated with ABL/PI5P (n = 16) or vehicle (n = 16), as described in the *Materials and Methods* section. At day 6 postinfection, mice were sacrificed, BALF was collected, and lungs were excised, homogenized, and plated onto TSA to determine bacterial burden. Counts of total number of cells **(A)**, neutrophils **(B)**, and macrophages **(C)** were performed in BALF. **(D)** The bacterial burden and was assessed by CFU assay. Data are shown as mean values + standard error. The data are pooled from two independent experiments. Statistical analysis was performed by using two-sided Mann–Whitney test. Statistical significance is indicated: *p < 0.05. Outlier data, identified by Grubbs’ test, were excluded by the analysis.

We wanted to test in an *in vivo* model the functional consequences of the *in vitro* observed anti-inflammatory functions of ABL/PI5P in addition to the promotion of intracellular killing of pathogens. This is particularly interesting since massive neutrophil infiltration is the main cause of chronic damage to the epithelial lung structure in the CF lung (30). Thus, we tested the efficacy of ABL/PI5P administrated by Penn-Century MicroSprayer^®^ Aerosoliser in mice, 5** **min after infection with MDR *P. aeruginosa* embedded in agar beads. An evaluation of the inflammatory response and bacterial burden in lung and in BALFs was considered as read-out measures of ABL/PI5P treatment efficacy. Results showed a significant reduction of both KC and MIP-2 (**Figures 6A, B** **)** and no significant variations in the levels of TNF-α and MCP-1 (**Figures 6C, D** **)** in the lungs of ABL/PI5P-treated mice in comparison with vehicle-treated mice. Results also showed a significant reduction in neutrophil count in BALF (**Figure 7B** **)** of ABL/PI5P-treated mice in comparison with vehicle-treated mice. A reduction, although not significant, of BALF total cells (**Figure 7A**) and macrophages (**Figure 7C**) was observed. Of note, the significant reduction in BALF neutrophils observed in ABL/PI5P-treated mice did not significantly interfere with pulmonary bacterial burden (**Figure 7D**).

## Discussion

CF is a genetic disorder that leads to a progressive dysfunction of lung activity by predisposing patients to colonization by opportunistic bacterial pathogens. Infections caused by *P. aeruginosa*, particularly because of the emergence of MDR strains, represent the major cause of morbidity and mortality in CF patients (31). These evidences highlight the urgency to develop novel therapeutic approaches, which may contribute to the control of MDR pathogens, including *P. aeruginosa*. Phagocytosis and intracellular killing of extracellular pathogens are the most important effector mechanisms of innate immune cells that can be hampered in CF patients (26). Hence, strategies aimed at improving the capacity of lung resident innate immune cells to phagocytose and kill pathogens may represent a promising host-directed approach to combat bacterial lung infections in CF patients.

In the present manuscript, we show that ABLs carrying PI5Ps are able to increase, both *in vitro* and *ex vivo*, the capacity of INH172-treated and CF macrophages to internalize and kill MDR strains of *P. aeruginosa*. Moreover, in a murine model of *in vivo* *P. aeruginosa* infection, we show that ABLs carrying PI5Ps are capable of reducing neutrophil recruitment and lung inflammation, without promoting bacterial growth. In particular, we show that treatment with ABL/PI5P enhance nonopsonic *P. aeruginosa* phagocytosis in CF and INH172-treated macrophages. Several not mutually exclusive mechanisms may explain this observation. PI5P may promote actin dynamics and bacterial phagocytosis i) *via* recruitment and activation of the exchange factor Tiam1 and Rac1 (14), ii) by directly activating PI3K/Akt signaling pathway (32), or iii) by participating as substrate to the PI(4,5)P_2_ production (33), which may directly induce membrane remodeling (34) or be converted, by means of phosphoinositide 3-kinase (PI3K), in 3,4,5-tris phosphate [PI(3,4,5)P_3_], which in turn is able to activate Akt signaling pathway (35).

We then showed that ABL/PI5P treatment restores intracellular acidification and ROS production of human macrophages, whose CFTR was pharmacologically inhibited. Following phagocytosis, phagosome maturation requires the sequential interaction with early endosomes, late endosomes, and ultimately, with lysosomes, leading to the generation of a highly microbiocidal organelle called phagolysosome. In pharmacologically inhibited- or CF-macrophages, the altered CFTR function leads to a limited phagosome acidification because of the unbalanced Cl^−^ ion distribution, which alters phagolysosome maturation and causes a defective intracellular bacterial clearance (8, 36). Together, our data indicate that ABL/PI5P treatment may rescue the impaired bactericidal mechanisms of macrophages with dysfunctional CFTR by restoring phagosome acidification and enhancing ROS production. Finally, the effect was specific to PI5P, as ABL loaded with PI3P, a second lipid messenger involved in membrane trafficking and autophagy (12), did not result in any modulation of intracellular *P. aeruginosa* killing (18).

The *ex vivo* analysis of MDM from CF patients indicated the presence of two groups of patients that we classified as “impaired” or “controller,” based on their different capability to control *in vitro* *P. aeruginosa* infection (bacterial replication index >1 or <1, respectively). It has been reported that host-genotypic traits have a critical role in the outcome of *P. aeruginosa* infection (37). In particular, the host susceptibility and the severity of infections caused by *P. aeruginosa* also depend upon a wide complex arrangement of genes, which is highly variable among immunocompromised individuals, including CF patients (38). Changes in clinical disease signs are mostly dependent on secondary gene variants that affect the outcome of the infection. These genes are identified as “modifier genes,” some of which play a role in innate immune response (39–41). Importantly, we observed that ABL/PI5P *ex vivo* treatment of macrophages induced a significant intracellular bacterial killing in the “impaired” group, highlighting the immunostimulant properties of ABL/PI5P, which restores the dysfunctional CF bactericidal response. On the contrary, the same treatment did not further increase the intracellular *P. aeruginosa* killing of macrophages from the “controller” group or from functional MDM by healthy donors. In agreement with these observations *in vitro*, we did not observe variations in terms of pulmonary bacterial burden in an *in vivo* model of *P. aeruginosa* chronic infection in immunocompetent mice. Together, these data support the hypothesis that ABL/PI5P treatment has no general and broad-spectrum immunoenhancing effect, but it is endowed with the potential to rescue impaired microbicidal innate immune function.

Airway inflammation is a hallmark of CF disease that leads to the decline in lung function (26) and is characterized by elevated levels of NF-κB activation and proinflammatory cytokine and chemokine production (30), resulting in chronic inflammation, neutrophil recruitment, and progressive airway destruction. It is still a matter of debate on whether excessive inflammation in CF is the result of either underlying chronic bacterial infection(s) in the lungs or of exaggerated NF-κB signaling (42). Results reported herein show that the levels of NF-κB activation increase in macrophages following *P. aeruginosa* infection, and such an increase is significantly higher following pharmacological inhibition of CFTR, both in uninfected and in infected macrophages in comparison with the control cells. However, despite higher basal NF-κB activation in the cells with pharmacologically inhibited CFTR, differences in TNF-α, IL-1β, IL-6 levels were observed in *P. aeruginosa-*infected macrophages only, suggesting that the presence of the pathogen is necessary to NF-κB-dependent proinflammatory cytokine production. These results support the hypothesis of a higher, NF-κB dependent, predisposition to a hyperinflammatory response by the macrophages with dysfunctional CFTR, which requires the presence of bacterial pathogens to over-express proinflammatory cytokines (30).

PS exposure at the outer surface of the cell membrane is a physiologically relevant signal for phagocytic cells, for which it represents the “eat me” signal provided by apoptotic bodies generated by cells undergoing apoptosis (43). This process is an anti-inflammatory/tolerogenic signal with immunomodulatory properties (44), which have been previously exploited for the treatment of autoimmune diseases (45). Furthermore, PI5P is involved in the activation of PI3K/Akt pathway that is crucial in restricting proinflammatory and promoting anti-inflammatory response (32, 46). The results reported herein support the anti-inflammatory and protolerogenic role of PS and PI5P even when they are delivered as a single liposome formulation. Based on these *in vitro* experimental results, we moved to the *in vivo* murine model of chronic *P. aeruginosa* infection and assessed the effects of ABL/PI5P treatment in terms of lung KC, MIP-2, JE and TNF-α production, leukocyte infiltrates, and pulmonary bacterial burden. Results show that in ABL-/PI5P-treated mice, the number of BALF neutrophils was significantly reduced, and such reduction paralleled with KC and MIP-2 levels, whereas any reduction of TNF-α and JE levels was not observed. The different results obtained following *in vitro* and *in vivo* infection, in terms of TNF-α production, may be explained by the activation of different cell types, such as antigen-specific Th1, Th17, and Th22 cells that may be involved and recruited to the lung during *in vivo* infections (47). Anti-inflammatory therapies, such as corticosteroids or biotechnologicals, may cause immunosuppression, which in turn is associated with the emergence of latent or opportunistic infections, and for this reason, they are often administered in combination with antibiotics (48). A clinical study to investigate the leukotriene B(4) (LTB(4)-receptor antagonist BIIL284 in CF patients was prematurely terminated due to a significant increased risk of adverse pulmonary events (49). Subsequent *in vivo* models showed that decreased airway neutrophils induced lung proliferation and severe bacteremia in a murine model of *P. aeruginosa* lung infection (50), indicating that strategies that interfere with neutrophil mechanisms have to be implemented with great caution. Of note, the reduction in inflammatory reactions in the lung of infected mice treated with ABL/PI5P was not associated with a significant increase in bacterial burden, suggesting that the *in vivo* administration of ABL/PI5P, by activating the macrophage component, may compensate for the reduction in neutrophil response and may have a therapeutic value also in critical conditions such as neutropenia.

Altogether, our data support the possibility that PI5P conveyed by ABL represents a novel therapeutic strategy devoid of immunosuppressive side effects, aimed at improving the efficiency of phagocytosis of mononuclear phagocytes and at reducing the damage of chronic inflammation. In conclusion, the ABL-/PI5P-based immunomodulatory strategy may represent an additional therapeutic tool in the fight against MDR opportunistic pathogens, such as *P. aeruginosa*, with the added value of the capacity to reduce the hyperinflammatory reactions in chronic lung infections that are particularly invalidating in CF patients.

## Data Availability Statement

All datasets generated for this study are included in the article/**Supplementary Material**.

## Ethics Statement

The studies involving human participants were reviewed and approved by Ethics Committee of “Bambino Gesù” Children’s Hospital, Rome, Italy. Ethics approval #738/2017. Written informed consent to participate in this study was provided by the participants’ legal guardian/next of kin. The animal study was reviewed and approved by Institutional Animal Care and Use Committee (IACUC) #733.

## Author Contributions

VL, RN, AB, and MF contributed to the conception and design of the study. NP, FDS, AR, SR, IDF, AH, and FC contributed to data acquisition. NP, MMDA, RN, AB, and MF participated in data analysis and manuscript writing. All authors contributed to the article and approved the submitted version.

## Funding

Research was supported by i) the Horizon 2020 Programme of European Commission, grant “EMI-TB”; Eliciting Mucosal Immunity against Tuberculosis—grant # 643558; ii) the Italian Cystic Fibrosis Research Foundation, FFC #14/2017, FFC#19/2019, and CFAaCore; iii) the Italian Foundation for multiple sclerosis, grant #2016/R/22; and iv) Regione Lazio, grant # E56C18000460002.

## Conflict of Interest

The authors declare that the research was conducted in the absence of any commercial or financial relationships that could be construed as a potential conflict of interest.

## References

[1] Castellani C Assael BM Cystic fibrosis: a clinical view. Cell Mol Life Sci (2017) 74:129–40. 10.1007/s00018-016-2393-9 PMC11107741 27709245

[2] Perez A Issler A Cotton CU Kelley TJ Verkman AS Davis PB CFTR inhibition mimics the cystic fibrosis inflammatory profile. Am J Physiol Lung Cell Mol Physiol (2007) 292:383–95. 10.1152/ajplung.00403.2005 16920886

[3] Ratjen F Döring G Cystic Fibrosis. Lancet (2003) 361:681–9. 10.1016/S0140-6736(03)12567-6 12606185

[4] Ferrari E Monzani R Villella VR Esposito S Saluzzo F Rossin F Cysteamine re-establishes the clearance of Pseudomonas *P. aeruginosa* by macrophages bearing the cystic fibrosis-relevant F508del-CFTR mutation. Cell Death Dis (2017) 8:e2544. 10.1038/cddis.2016.476 28079883 PMC5386380

[5] Cystic fibrosis foundation patient registry Annual data report. Bruce C Marshall MD, editors. Bethesda, Maryland: Cystic Fibrosis Foundation (2018).

[6] O’ Sullivan BP Freedman SD Cystic Fibrosis. Lancet (2009) 373:1891–904. 10.1016/S0140-6736(09)60327-5 19403164

[7] Donnelly LE Barnes PJ Defective phagocytosis in airways disease. Chest (2012) 141:1055–62. 10.1378/chest.11-2348 22474147

[8] Di A Brown ME Deriy LV Li C Szeto FL Chen Y CFTR regulates phagosome acidification in macrophages and alters bactericidal activity. Nat Cell Biol (2006) 8:933–44. 10.1038/ncb1456 16921366

[9] Yeung T Ozdamar B Paroutis P Grinstein S Lipid metabolism and dynamics during phagocytosis. Curr Opin Cell Biol (2006) 18:429–37. 10.1016/j.ceb.2006.06.006 16781133

[10] Steinberg BE Grinstein S Pathogen destruction versus intracellular survival: the role of lipids as phagosomal fate determinants. J Clin Invest (2008) 118:2002–11. 10.1172/JCI35433 PMC2396921 18523652

[11] Nisini R Poerio N Mariotti S De Santis F Fraziano M The Multirole of Liposomes in Therapy and Prevention of Infectious Diseases. Front Immunol (2018) 9:155. 10.3389/fimmu.2018.00155 29459867 PMC5807682

[12] De Craene J Bertazzi DL Bär S Friant S Phosphoinositides, major actors in membrane traffcking and lipid signaling pathways. Int J Mol Sci (2017) 18:634. 10.3390/ijms18030634 PMC5372647 28294977

[13] Oppelt A Lobert VH Haglund K Mackey AM Rameh LE Liestøl K Production of phosphatidylinositol 5-phosphate via PIKfyve and MTMR3 regulates cell migration. EMBO Rep (2013) 14:57–64. 10.1038/embor.2012.183 23154468 PMC3537138

[14] Viaud J Lagarrigue F Ramel D Allart S Chicanne G Ceccato L Phosphatidylinositol 5-phosphate regulates invasion through binding and activation of Tiam1. Nat Commun (2014) 5:4080. 10.1038/ncomms5080 24905281

[15] Yeung T Grinstein S Lipid signaling and the modulation of surface charge during phagocytosis. Immunol Rev (2007) 219:17–36. 10.1111/j.1600-065X.2007.00546.x 17850479

[16] Schaletzky J Dove SK Short B Lorenzo O Clague MJ Barr FA Phosphatidylinositol-5-phosphate activation and conserved substrate specificity of the myotubularin phosphatidylinositol 3-phosphatases. Curr Biol (2003) 13:504–9. 10.1016/s0960-9822(03)00132-5 12646134

[17] Vicinanza M Korolchuk VI Ashkenazi A Puri C Menzies FM Clarke JH PI(5)P regulates autophagosome biogenesis. Mol Cell (2015) 57:219–34. 10.1016/j.molcel.2014.12.007 PMC4306530 25578879

[18] Greco E Quintiliani G Santucci MB Serafino A Ciccaglione AR Marcantonio C Janus-faced liposomes enhance antimicrobial innate immune response in Mycobacterium tuberculosis infection. Proc Natl Acad Sci USA (2012) 109:E1360–8. 10.1073/pnas.1200484109 PMC3361443 22538807

[19] Poerio N Bugli F Taus F Santucci MB Rodolfo C Cecconi F Liposomes loaded with bioactive lipids enhance antibacterial innate immunity irrespective of drug resistance. Sci Rep (2017) 7:45120. 10.1038/srep45120 28345623 PMC5366871

[20] Jeukens J Boyle B Bianconi I Kukavica-Ibrulj I Tümmler B Bragonzi A Complete Genome Sequence of Persistent Cystic Fibrosis Isolate Pseudomonas *P. aeruginosa* Strain RP73. Genome Announc (2013) 1(4):e00568-13. 10.1128/genomeA.00568-13 23908295 PMC3731849

[21] Facchini M De Fino I Riva C Bragonzi A Long term chronic Pseudomonas *P. aeruginosa* airway infection in mice. JoVE (2014) 85:51019. 10.3791/51019 PMC4151694 24686327

[22] Cigana C Ranucci S Rossi A De Fino I Melessike M Bragonzi A Antibiotic efficacy varies based on the infection model and treatment regimen for Pseudomonas aeruginosa. Eur Respir J (2019) 55(3):1802456. 10.1183/13993003.02456-2018 PMC7057181 31624114

[23] Zhang Y Li X Grassmé H Döring G Gulbins E Alterations in ceramide concentration and pH determine the release of reactive oxygen species by Cftr-deficient macrophages on infection. J Immunol (2010) 184:5104–11. 10.4049/jimmunol.0902851 20351190

[24] Cigana C Bernardini F Facchini M Alcalá-Franco B Riva C De Fino I Efficacy of the Novel Antibiotic POL7001 in Preclinical Models of Pseudomonas *P. aeruginosa* Pneumonia. Antimicrob Agents Chemother (2016) 60(8):4991–5000. 10.1128/AAC.00390-16 27297477 PMC4958219

[25] Zhang S Shrestha CL Kopp BT Cystic fibrosis transmembrane conductance regulator (CFTR) modulators have differential effects on cystic fibrosis macrophage function. Sci Rep (2018) 8:17066. 10.1038/s41598-018-35151-7 30459435 PMC6244248

[26] Lévêque M Le Trionnaire S Del Porto P Martin-Chouly C The impact of impaired macrophage functions in cystic fibrosis disease progression. J Cyst Fibros (2017) 16:443–53. 10.1016/j.jcf.2016.10.011 27856165

[27] Sallenave JM Phagocytic and signaling innate immune receptors: are they dysregulated in cystic fibrosis in the fight against Pseudomonas aeruginosa? Int J Biochem Cell Biol (2014) 52:103–7. 10.1016/j.biocel.2014.01.013 24508137

[28] Nunes P Demaurex N Dinauer MC Regulation of the NADPH oxidase and associated ion fluxes during phagocytosis. Traffic (2013) 14:1118–113. 10.1111/tra.12115 23980663

[29] Malhotra S Hayes D Jr Wozniak DJ Cystic Fibrosis and Pseudomonas aeruginosa: the Host-Microbe Interface. Clin Microbiol Rev (2019) 32(3):e00138-18. 10.1128/CMR.00138-18 31142499 PMC6589863

[30] Cohen-Cymberknoh M Kerem E Ferkol T Elizur A Airway inflammation in cystic fibrosis: molecular mechanisms and clinical implications. Thorax (2013) 68:1157–62. 10.1136/thoraxjnl-2013-203204 23704228

[31] Stefani S Campana S Cariani L Carnovale V Colombo C Lleo MM Relevance of multidrug-resistant Pseudomonas *P. aeruginosa* infections in cystic fibrosis. Int J Med Microbiol (2017) 6:353–62. 10.1016/j.ijmm.2017.07.004 28754426

[32] Pendaries C Tronchère H Arbibe L Mounier J Gozani O Cantley L PtdIns5P activates the host cell PI3-kinase/Akt pathway during Shigella flexneri infection. EMBO J (2006) 25:1024–34. 10.1038/sj.emboj.7601001 PMC1409730 16482216

[33] Hasegawa J Iwamoto R Otomo T Nezu A Hamasaki M Yoshimori T Autophagosome-lysosome fusion in neurons requires INPP5E, a protein associated with Joubert syndrome. EMBO J (2016) 35:1853–67. 10.15252/embj.201593148 PMC5007553 27340123

[34] Janmey PA Lindberg U Cytoskeletal regulation: rich in lipids. Nat Rev Mol Cell Biol (2004) 5:658–66. 10.1038/nrm1434 15366709

[35] Lovewell RR Hayes SM O’Toole GA Berwin B Pseudomonas *P. aeruginosa* flagellar motility activates the phagocyte PI3K/Akt pathway to induce phagocytic engulfment. Am J Physiol Lung Cell Mol Physiol (2014) 306:698–707. 10.1152/ajplung.00319.2013 PMC3962627 24487390

[36] Del Porto P Cifani N Guarnieri S Di Domenico EG Mariggiò MA Spadaro F Dysfunctional CFTR alters the bactericidal activity of human macrophages against Pseudomonas aeruginosa. PloS One (2011) 6:e19970. 10.1371/journal.pone.0019970 21625641 PMC3097223

[37] Lorè NI Iraqi FA Bragonzi A Host genetic diversity influences the severity of Pseudomonas *P. aeruginosa* pneumonia in the Collaborative Cross mice. BMC Genet (2015) 16:106. 10.1186/s12863-015-0260-6 26310945 PMC4551369

[38] Gellatly SL Hancock RE Pseudomonas aeruginosa: new insights into pathogenesis and host defenses. Pathog Dis (2013) 67:159–73. 10.1111/2049-632X.12033 23620179

[39] Weiler CA Drumm ML Genetic influences on cystic fibrosis lung disease severity. Front Pharmacol (2013) 4:40. 10.3389/fphar.2013.00040 23630497 PMC3632778

[40] Bruscia EM Bonfield TL Cystic Fibrosis Lung Immunity: The Role of the Macrophage. J Innate Immun (2016) 8:550–63. 10.1159/000446825 PMC5089923 27336915

[41] Cutting GR Cystic fibrosis genetics: from molecular understanding to clinical application. Nat Rev Genet (2015) 16:45–56. 10.1038/nrg3849 25404111 PMC4364438

[42] Roesch EA Nichols DP Chmiel JF Inflammation in cystic fibrosis: An update. Pediatr Pulmonol (2018) 53:S30–50. 10.1002/ppul.24129 29999593

[43] Segawa K Nagata S An Apoptotic ‘Eat Me’ Signal: Phosphatidylserine Exposure. Trends Cell Biol (2015) 25:639–50. 10.1016/j.tcb.2015.08.003 26437594

[44] Birge RB Ucker DS Innate apoptotic immunity: The calming touch of death. Cell Death Differ (2008) 15:1096–102. 10.1038/cdd.2008.58 18451871

[45] Pujol-Autonell I Mansilla MJ Rodriguez-Fernandez S Cano-Sarabia M Navarro-Barriuso J Ampudia RM Liposome-based immunotherapy against autoimmune diseases: therapeutic effect on multiple sclerosis. Nanomedicine (Lond) (2017) 12:1231–42. 10.2217/nnm-2016-0410 28593827

[46] Vergadi E Ieronymaki E Lyroni K Vaporidi K Tsatsanis C Akt Signaling Pathway in Macrophage Activation and M1/M2 Polarization. J Immunol (2017) 198:1006–14. 10.4049/jimmunol.1601515 28115590

[47] Bayes HK Bicknell S MacGregor G Evans TJ T helper cell subsets specific for Pseudomonas *P. aeruginosa* *in* healthy individuals and patients with cystic fibrosis. PloS One (2014) 9:e90263. 10.1371/journal.pone.0090263 24587305 PMC3937364

[48] Vozoris NT Seemangal J Batt J Prevalence, screening and treatment of latent tuberculosis among oral corticosteroid recipients. Eur Respir J (2014) 44:1373–5. 10.1183/09031936.00076714 24969656

[49] Konstan M Döring G Heltshe SL Lands LC Hilliard KA Koker P Investigators and Coordinators of BI Trial 543.45. A randomized double blind, placebo controlled phase 2 trial of BIIL 284 BS (an LTB4 receptor antagonist) for the treatment of lung disease in children and adults with cystic fibrosis. J Cyst Fibros (2014) 13:148–55. 10.1016/j.jcf.2013.12.009 PMC4755340 24440167

[50] Döring G Bragonzi A Paroni M Aktürk FF Cigana C Schmidt A BIIL 284 reduces neutrophil numbers but increases P. aeruginosa bacteremia and inflammation in mouse lungs. J Cyst Fibros (2014) 13:156–63. 10.1016/j.jcf.2013.10.007 PMC4163938 24183915

