# Supplementary material for: Liposomes Loaded With Phosphatidylinositol 5-Phosphate Improve the Antimicrobial Response to Pseudomonas aeruginosa in Impaired Macrophages From Cystic Fibrosis Patients and Limit Airway Inflammatory Response
Source: Front Immunol. 2020 Oct 2;11:532225. doi: 10.3389/fimmu.2020.532225 (PMC7562816; doi:10.3389/fimmu.2020.532225)
Supplement: Supplementary file 1 [file DataSheet_1.pdf]

Supplementary Material

Supplementary Methods

Liposome preparation. Liposomes were produced as previously described [18-19]. Briefly, the inner monolayer lipids were composed by either 1,2-dioleoyl-*sn*-glycero-3-phospho-(1′-myo-inositol-5′-phosphate) (PI5P, Avanti Polar Lipids) or L-α-phosphatidylserine (PS, Avanti Polar Lipids), and were suspended in anhydrous dodecane (Sigma) at the concentration of 0.05 mg/ml. Both PS and PI5P were also used as outer monolayer lipid and were added to a 99:1 dodecane:silicone solution to obtain a final concentration of 0.05 mg/ml. Liposomes were prepared by adding 2 ml of outer monolayer lipid suspension over 3 ml of cell culture medium (for *in vitro* experiments) or saline (for *in vivo* experiments). Finally, 100 µl of the inner monolayer lipid suspensions were added over 2 ml lipid phase and the samples were centrifuged at 120 g for 10 minutes. After the centrifugation, liposomes were collected in the aqueous phase using a 5 ml syringe with a 16-gauge stainless steel needle. The following liposome formulations were produced: i) liposomes with PS at the outer membrane leaflet and PI5P at the inner membrane leaflet (ABL/PI5P), ii) liposomes with PI5P both at the outer and inner membrane leaflet (PI5P/PI5P) or iii) liposomes with PS both at the outer and inner membrane leaflet (ABL/PS). Liposomes were then quantified by a flow cytometer FACSCalibur (Becton Dickinson), allowing quantification of monodispersed vesicles >0.2 µm in diameter.

Cell culture. Human pro-monocytic THP-1 leukemia cell line was supplied by European Collection of Cell Culture, grown in RPMI 1640 containing fetal bovine serum (10%), gentamycin (5 µg/ml), **L-**

glutammine (2 mM), nonessential amino acids (1 mM), sodium pyruvate (1 mM) and cultured in 75 cm^2 polystyrene flasks. Before experiments, cells (2×10^5 per well) were seeded in 96-well plates and cells were induced to differentiate by stimulation for 72 hours with Phorbol 12-Myristate 13-Acetate (PMA) (20 ng/ml), and used as a model of human macrophages (dTHP-1).

Primary monocyte derived macrophages (MDM) were isolated from buffy coats by healthy blood donors, attending at the Blood Transfusion Unit of Policlinico "Umberto I" in Rome, Italy, and prepared as previously described [Poerio N. et al. Sci Rep. 2017;7:45120]. Briefly, peripheral blood mononuclear cells were isolated by healthy donors or Cystic Fibrosis (CF) patients and monocytes were separated, by using anti-CD14 monoclonal antibodies conjugated to magnetic microbeads (Miltenyi Biotec), according to manufacturer's instructions. Monocytes were then suspended in complete medium and incubated for a further 5 days in 96-well plates at the concentration of 10^6 cells/ml in the presence of M-CSF (50 ng/mL) (Miltenyi Biotec) to get differentiated macrophages.

Bacteria. Multidrug-resistant (MDR) *Pseudomonas aeruginosa* strains **(**ATCC® BAA-2108, ATCC® BAA-2111, ATCC® BAA-2112, ATCC® BAA-2113), was used. The different MDR *P. aeruginosa* single colonies were collected by streaking on Trypticase soy agar (TSA) (BD Difco™) and suspended in 15 ml of Trypticase soy broth (TSB) (BD Difco™). Bacteria were grown in Erlenmeyer flask at 37°C under stirring for 18 hours their growth was monitored by measuring the optical density at the wavelength of 600 nm by Varioskan LUX Multimode Microplate Reader (Thermo Fisher Scientific). BAA-2108, BAA-2111, BAA-2112, BAA-2113 were stored at - 80°C until use after suspension in TSB and 30% glycerol.

Evaluation of *in vitro* bacterial intracellular growth. To assess intracellular bacterial growth, dTHP-1 cell or MDM from healthy donors were distributed in 96 well plates at the concentration of $2x10^5$ cells/well and were infected with BAA-2108, BAA-2111, BAA-2112 or BAA-2113, for 1 hour at 37°C at a MOI of 30 or 10 in the presence or absence of INH172, used at the concentration of 10 µM. Thereafter, extracellular bacilli were killed by 1 hour incubation with 400 µg/ml amikacin. Cells were then washed and incubated with ABL/PI5P, added to a ratio of 1 to 1 (ABL:MDM) for further 2 hours, in the presence or absence of INH172. Finally, cells were lysed with 1% deoxycholate (Sigma), samples diluted in PBS-tween 80 and CFU quantified by plating bacilli in triplicate on TSA.

Evaluation of intraphagosomal acidification. Intraphagosomal acidification was assessed by using microbeads 1 µm (Sigma-Aldrich) labelled with the pH sensitive dye N-hydroxysuccin-imidyl 5-(and 6-)-carboxyfluorescein (NHS-CF) (100 µg/ml, Sigma), as described [Pethe K. et al. Proc Natl Acad Sci U S A. 2004; 101(37):13642-7].In particular, MDM were pre-treated or not for 1 hour with INH172 (10 µM), and exposed with NHS labelled microbeads, for 1 hour at 37 ° C at a ratio of 5:1 in the presence or absence of 10 µM of the CFTR inhibitor INH172 (Sigma). Cells were then washed and incubated for further 90 minutes in the presence or absence of INH172 with ABL/PI5P, added to a ratio of 1 to 1 (ABL:MDM). The intensity of fluorescence was determined at an excitation wavelength of 492 nm and emission wavelength of 517 nm, by the use of a Varioskan LUX Multimode Microplate Reader (Thermo Fisher Scientific).

Supplementary Figures

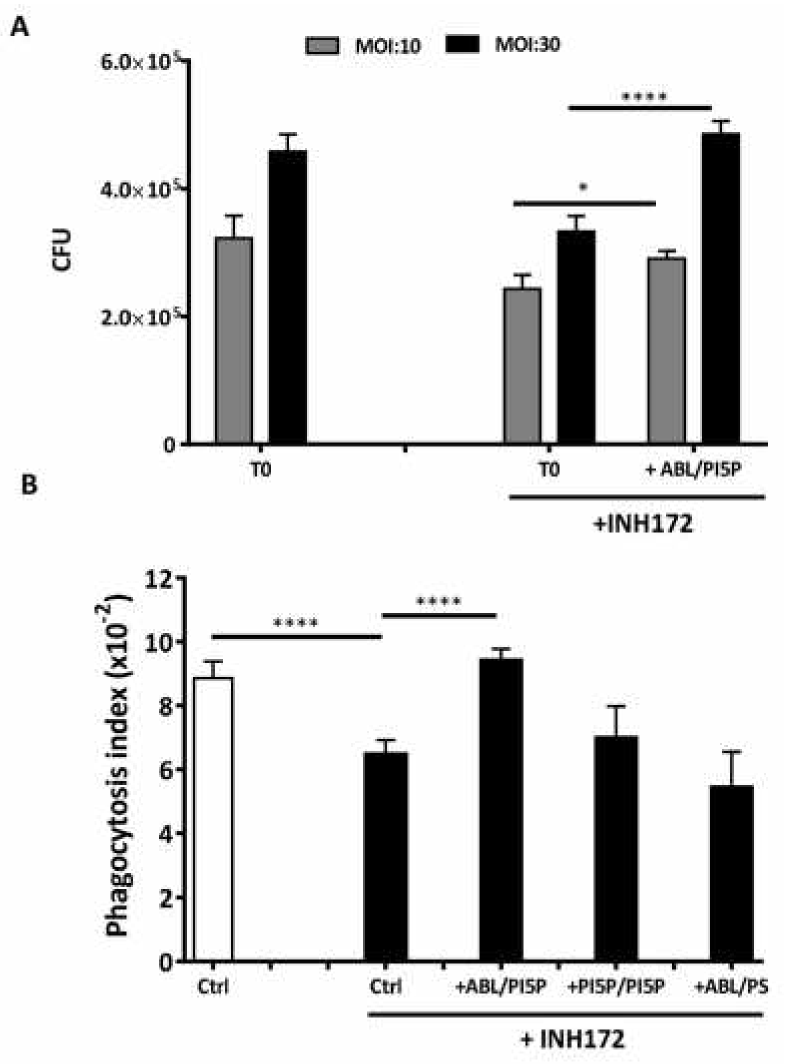

Fig. S1. ABL/PI5P improves MDR *P. aeruginosa* internalization in dTHP1 cells with pharmacologically inhibited CFTR. dTHP-1 cells, treated or not with INH172, were stimulated or not with ABL/PI5P, PI5P/PI5P or ABL/PS (used as controls) for 30 minutes at 37°C before infection. Cells were then infected with MDR *P. aeruginosa* (BAA-2113 strain) at the MOI of 10 (A) or 30 (A and B). The bacterial uptake was quantified by CFU assay, and indicated as: (A) CFUs obtained immediately after the infection (T0); (B) phagocytosis index, calculated as the ratio between the CFUs obtained immediately after the infection and the inoculum. The results are shown as mean + standard deviation of the values obtained from the triplicate of each condition. *p<0,05; **** p< 0,0001 by two-sided Student's t test.

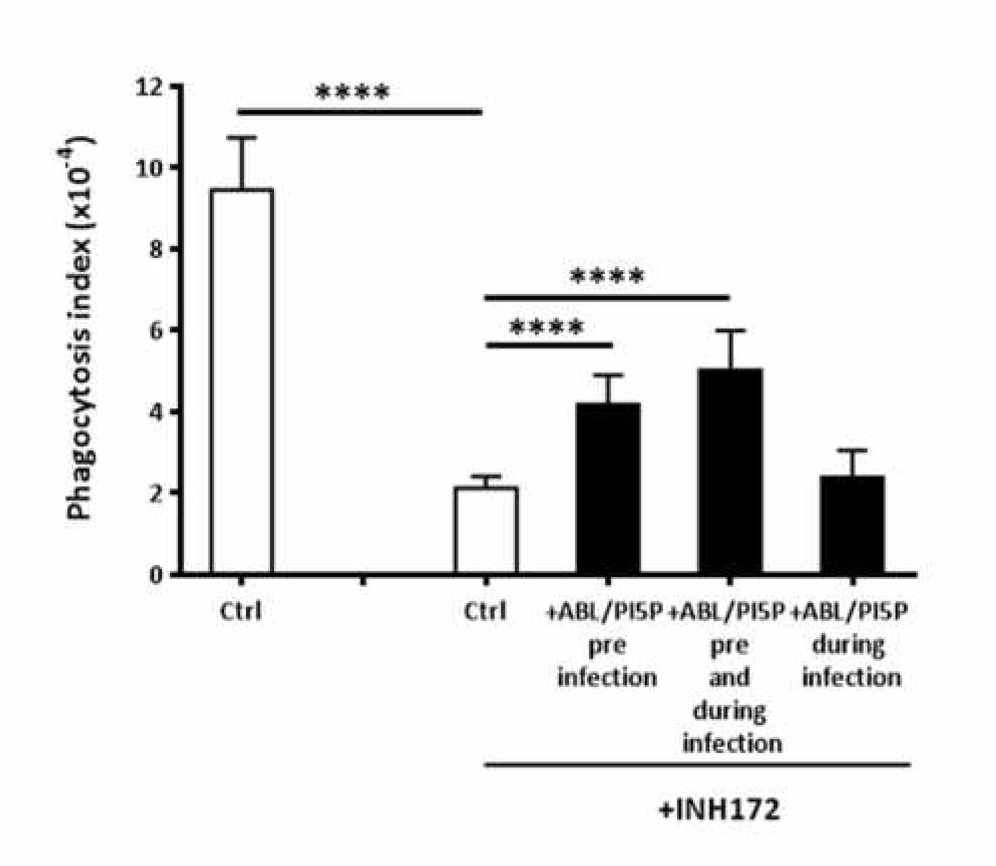

Fig. S2. ABL loaded with PI5P improve dysfunctional *P. aeruginosa* uptake in primary MDM with pharmacologically inhibited CFTR. Primary MDM from healthy donors, treated or not with INH172, were stimulated or not with ABL loaded with PI5P for 30 minutes at 37°C before infection (pre infection), and/or simultaneously to the infection (during infection). Cells were infected with MDR *P. aeruginosa* (BAA-2113 strain) at the MOI of 30. The bacterial uptake was quantified by CFU assay and indicated as phagocytosis index, calculated as the ratio between the CFUs obtained immediately after the infection and the inoculum. The results are shown as mean + standard deviation of the values obtained from the triplicate of each condition and are representative of experiments with cells by two different donors. **** p< 0,0001 by two-sided Student's t test.

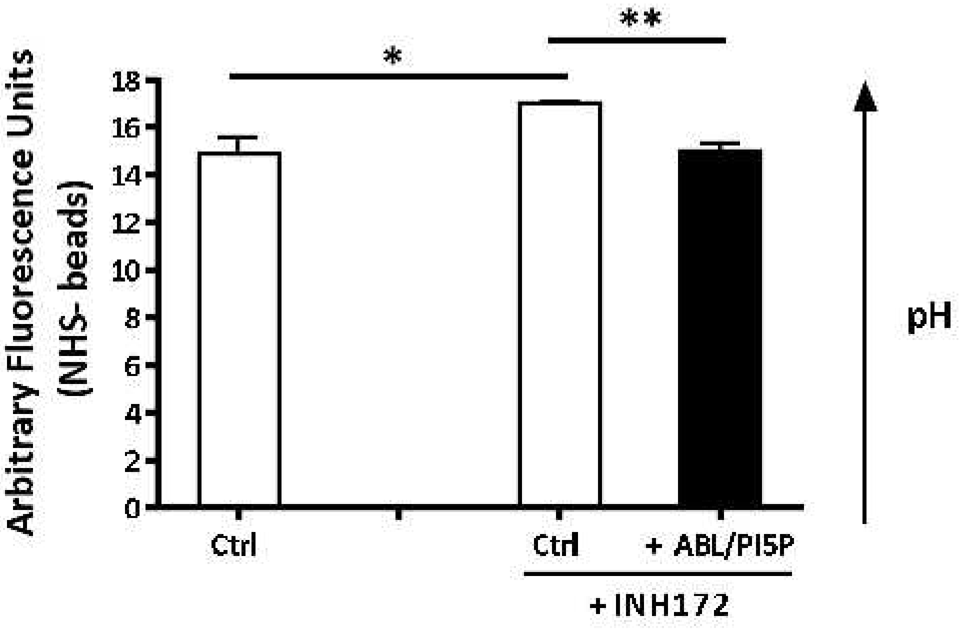

Fig. S3. ABL/PI5P promote intraphagosomal acidification in primary macrophages expressing a pharmacologically inhibited CFTR. MDM, treated or not with INH172, were exposed for 1 hour to NHS labelled microbeads. Intraphagosomal pH was assessed by fluorescence decrease of NHS after 90 minutes from the stimulation with ABL/PI5P. Results are shown as mean + standard deviation of the values obtained from triplicate cultures and are representative of experiments with cells by two different donors. * p< 0,05 ; ** p< 0,01, in comparison with INH172-unstimulated cells and in comparison with untreated cells by one-sided Student's t test.

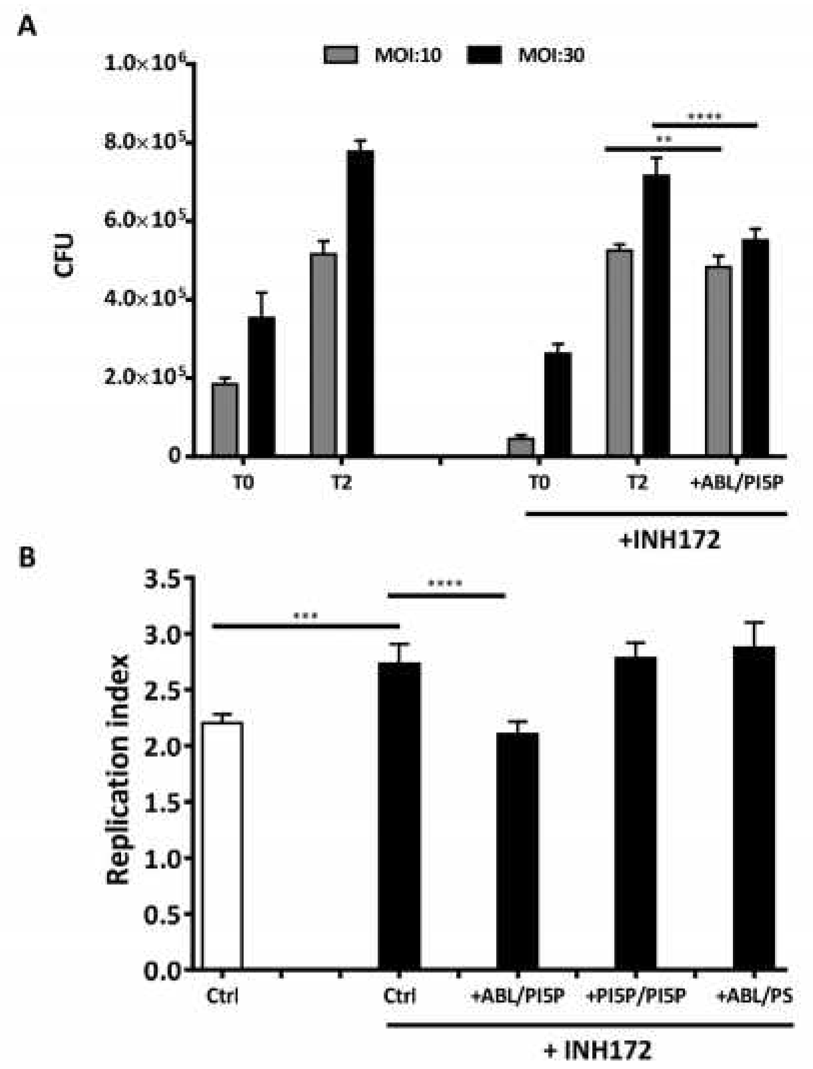

Fig. S4. ABL/PI5P improves MDR *P. aeruginosa* intracellular killing in dTHP1 cells with pharmacologically inhibited CFTR. dTHP-1 cells, treated or not with INH172, were infected with MDR *P. aeruginosa* (BAA-2113 strain) at the MOI of 10 (A) or 30 (A and B) and then stimulated for 2 hours with the ABL/PI5P, PI5P/PI5P or ABL/PS. Bacterial growth was assessed by CFU assay, and indicated as: (A) CFUs obtained immediately after the infection and CFUs obtained after two hours of infection are indicated as T0 and T2, respectively; (B) replication index, calculated as the ratio between the CFU obtained after two hours of infection, in the presence or absence of stimuli, and the CFU obtained before the addition of liposomes. The results are shown as mean + standard deviation of the values obtained from the triplicate of each condition. **p<0,01; ***p<0,001; **** p< 0,0001 by two-sided Student's t test.

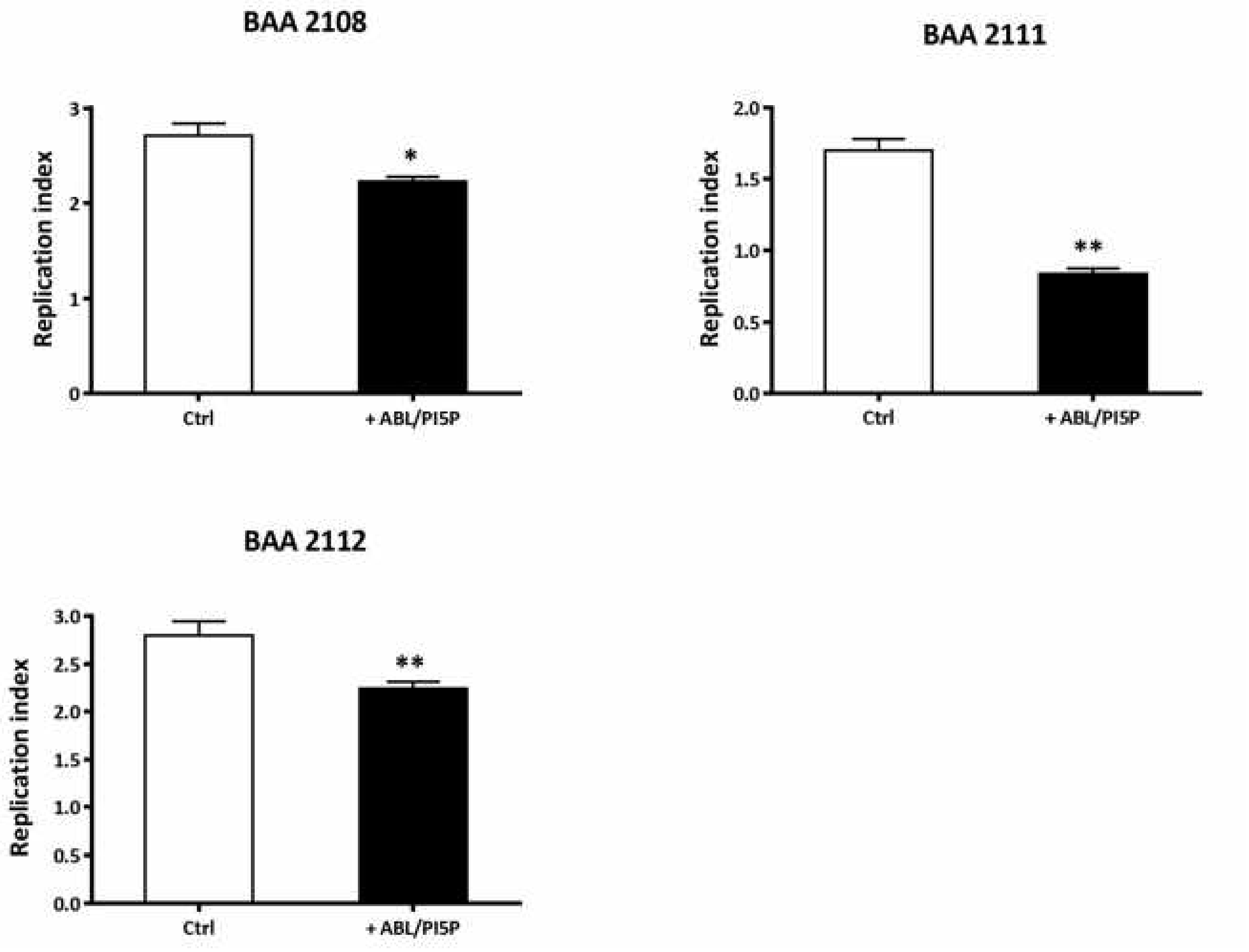

Fig. S5. ABL/PI5P promote intracellular bacterial killing in primary macrophages expressing a pharmacologically inhibited CFTR. MDM isolated from healthy donor were treated with INH172 and infected with MDR *P. aeruginosa* (BAA-2108, BAA-2111 or BAA-2112 strains) and then stimulated for 2 hours with the ABL/PI5P. Bacterial growth was assessed by CFU assay and replication index was calculated as the ratio between the CFU obtained after two hours of infection, in the presence or absence of ABL/PI5P, and the CFU obtained before the addition of liposomes. The results are shown as mean + standard deviation of the values obtained from the triplicate of each condition, * p< 0,05 ; ** p< 0,01 in comparison with unstimulated cells by one- sided Student's *t* test.

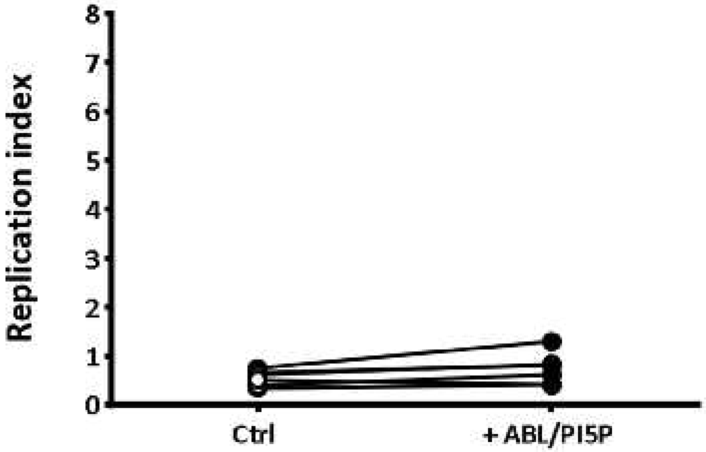

Fig. S6. ABL/PI5P does not modulate MDR *P. aeruginosa* intracellular bacterial killing in primary MDM. MDM isolated from healthy donor (n=6) were infected with MDR *P.aeruginosa* (BAA-2113 strain) and then stimulated for 2 hours with the ABL/PI5P. Bacterial growth was assessed by CFU assay and replication index was calculated as the ratio between the CFU obtained after two hours of infection, in the presence or absence of ABL/PI5P, and the CFU obtained before the addition of liposomes. Statistical analysis was performed by using two-sided Wilcoxon matched-pairs signed rank test. (p value = not significant)
